# The Effect of Long-Term Moderate Static Magnetic Field Exposure on Adult Female Mice

**DOI:** 10.3390/biology11111585

**Published:** 2022-10-28

**Authors:** Xingxing Yang, Biao Yu, Chao Song, Chuanlin Feng, Jing Zhang, Xinyu Wang, Guofeng Cheng, Rui Yang, Wei Wang, Yong Zhu

**Affiliations:** 1 School of Life Sciences, Hefei Normal University, Hefei 230601, China; 2 High Magnetic Field Laboratory, Hefei Institutes of Physical Science, Chinese Academy of Sciences, Hefei 230031, China

**Keywords:** static magnetic field, long-term, female, behavioral, antioxidant capacity

## Abstract

**Simple Summary:**

The data show that long-term 150 mT SMF exposure enhanced the motility and alleviated the mental status of adult female mice by modulating glucose metabolism and gut microbiota, increasing the level of beneficial flora and antioxidant capacity. This certificated that moderate intensity long-term SMF exposure has the potential to ameliorate the locomotive and exploratory capacity and negative mood associated with female growth.

**Abstract:**

Because of the high cost and safety of ultra-high magnetic resonance imaging (MRI), its application has certain limitations. Whereas 0.5–3 T MRI has been widely applied in hospitals, static magnetic fields (SMFs) have been shown to improve mice mental health and have anti-tumor potentials. Here, we compared the effects of the upward and downward 150 mT SMF groups with the sham group on C57BL/6J adult female mice. Locomotor and exploratory activity were also measured by behavioral tests, including the open field and elevated plus test. Additionally, physiology, pathology indicators and gut microbiota were examined. We found that 150 mT SMFs long-term exposure enhanced locomotive and exploratory activity of mice, especially the downward 150 mT SMF. Compared with the downward 150 mT SMF group, the movement speed and distance in the center area of the sham group were increased by 65.99% (*p* < 0.0001) and 68.58% (*p* = 0.0038), respectively. Moreover, compared to the sham group, downward 150 mT SMF increased the number of entrances to the center area by 67.0% (*p* = 0.0082) and time in the center area by 77.12% (*p* = 0.0054). Additionally, we observed that upward 150 mT SMF improved the number of follicles (~2.5 times, *p* = 0.0325) and uterine glands through increasing the total antioxidant capacity and reducing lipid peroxidation level in mice. Gut microbiome analysis showed that 150 mT SMFs long-term exposure improved the microbiota abundance (*Clostridium*, *Bifidobacterium*, *Ralstonia* and *Yaniella*) in the genus level, which may affect metabolism, anxiety and behavior in adult female mice. Our results demonstrated that 150 mT SMFs long-term exposure not only had good biosafety, but also improved athletic performance, emotion and the function of ovarian, uterine and gut microbiota abundance in adult female mice, which unraveled the potential of moderate long-term SMF exposure in clinical applications.

## 1. Introduction

With the widespread application and development of magnetic resonance imaging (MRI), the MRI in most hospitals is presently 1.5–3 T [1]. Since the static magnetic field of an MRI scanner is always on, MRI workers are always exposed to SMF. It is reported that in the course of a patient’s routine examination during a 1.5 T MRI, the level of exposure of nurses to SMF may vary from 0.5 mT to 180 mT, and sometimes the exposure intensity may exceed 500 mT when patients need more attention [2]. A study showed that the frequencies of adverse health effects such as headache, sleep problems and nervousness in MRI staffs were increased [3]. In fact, except for in MRIs, magnetic fields also have applications in conventional and alternative medicine; for example, Juhász found that inhomogeneous SMF (2.77 μT–192 mT) exposure reduced the erosive gastritis of patient [4], and researchers also found that sleeping on a 110 mT magnetic mattress pad for 16 weeks improved chronic pain and sleep disturbances experienced by patients with fibromyalgia [5]. In addition to mattresses, there are also magnetized insoles [6], magnetic necklaces [7] and so on, and with the widespread application of magnetic fields, the time and opportunity for moderate magnetic fields exposure has also increased significantly for humans; therefore, the public should be very concerned about the biosafety of static magnetic fields (SMF). Therefore, it is meaningful and necessary to explore the long-term effects of moderate SMF on healthy humans.

In our lives, when we face various stresses, we feel depressed or anxious about our perception or evaluation of the event that determines the subsequent reaction or coping behavior [8]. It was reported that depression and anxiety disorders affect women approximately twice as much as they affect men and occur predominately during a woman’s reproductive years [9]. Depression and anxiety are also related to physical activity, and increased depression and anxiety are often accompanied by decreased physical activity; however, increased athletics can alleviate depression [10]. There is some researches about the effects of SMF on a male’s mood or locomotive ability [11,12,13]; Tasić et al. found that after 30 days of 16 mT SMF exposure, male spontaneously hypertensive rats spend more time in the open arm and less time in the peripheral region, but no differences in climbing and mobility with control were found [14]. However, recent research found that total distance, surrounding distance, activity time, climbing and standing times of male mice were significantly decreased after 100 mT SMF 15 days (1 h/d) exposure, and resulted in anxiety-like behaviors [15]. High prevalence of psychological stress, anxiety and depression in women has been recognized in worldwide, but it is rarely seen for moderate intensity SMF exposure and only few reports concern the effects of high-intensity SMF exposure on females [16,17,18]. Except for gender differences, SMF intensity and exposure time are other reasons which led to inconsistent conclusions. For example, Kokoreva et al. found that after the exposure (3 h/d) of 1.6 T SMF for 1 day, 5 days and 15 days, the swimming time of mice (unknown gender) was prolonged and exercise tolerance was enhanced [11]. Ivan Tkac et al. found that 16.4 T SMF 4- or 8-week exposure (3 h/time, 2 times/week) did not affect cognition and motor function in female and male mice [19]. We found the SMF exposure time was short in the above research, and it is urgent to explore the biosafety and effect of long-term SMF exposure (continuous treat for several months) on females.

In this study, we explored the effects of upward and downward 150 mT SMFs long-term exposure in healthy female adult mice. Our results showed that downward 150 mT SMF effectively enhanced locomotive activity, and ameliorated depression and anxiety by possibly modulating glucose metabolism and gut microbiota in healthy female mice.

## 2. Materials and Methods

### 2.1. Animals and Static Magnetic Field

It is reported that mice attain sexual maturity at 8–12 weeks [20,21], and we purchased 18 eight-week-old female C57BL/6J mice from Gempharmatech (Nanjing, China). The mice were raised in a sterile environment with enough water and food. All protocols were approved by the Ethics and Humane Committee of Hefei Institute of Physical Sciences, Chinese Academy of Sciences (DWLL-2021-46), and strictly followed the National Institutes of Health Guide for the Care and Use of Laboratory Animals (NIH Publication No. 8023, revised 1978). Mice were divided into 3 groups randomly with 6 mice in each group. We recorded water and food intake, as well as weight weekly, and measured the fasting blood glucose (fast 12 h) monthly by blood glucose meters and blood glucose test strips (Roche, Shanghai, China).

**Figure 1:**
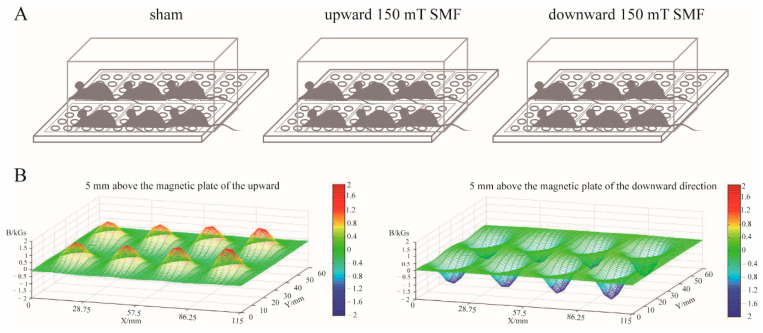
Magnetic field exposure system. (**A**) 150 mT magnetic plates were composed by 10 small magnetic blocks with the same magnetic pole (N or S). The cages were placed on the magnetic plate, and each cage contained 6 mice. (**B**) The magnetic flux intensity of 150 mT magnetic plates were about 150 mT at 5 mm above the plates.

The magnetic plate used in the mice experiments was composed of 10 small magnetic plates, and each small magnetic plate contained 8 magnets with a strength of 150 mT and which had the same polarity (N or S), thus forming the upward (N) or downward (S) magnetic plate. Mice from the 150 mT magnetic field groups were placed on the magnetic plate, and the sham group was placed on the non-magnetic plate for 18 weeks (24 h/d) until the mice were 6 months. From week 8 to week 26, the mice were equivalent to 20–30 years old in human years, which mirrors awoman’s reproductive age (https://resources.jax.org/therapeutic-area-neurobiology/whitepaper-aged-b6 accessed on 10 September 2022). The magnetic field flux intensity from the plate to the mice abdomens was reduced to 150 mT, so the upward and downward 150 mT SMFs were used in this paper (Figure 1).

### 2.2. Complete Blood Count and Blood Biochemistry Analysis

Blood samples were taken through the orbital venous plexus before mice were sacrificed. In total, 200 µL blood was taken and placed in the EP tube with or without EDTA-K_2_, and serum was collected by centrifugation when blood was placed at 4 °C for 20 min. All blood samples were sent to Service Biotechnology (Wuhan, China) on dry ice for immediate blood biochemistry analysis. Blood biochemistry mainly detected the level of aspartic transaminase (AST), alanine aminotransferase (ALT), total bilirubin (TBIL), serum creatinine (CREA), total cholesterol (TC), triglyceride (TG), high-density lipoprotein cholesterol (HDL-c), low-density lipoprotein cholesterol (LDL-c) and uric acid (UA). Mice were killed immediately after blood was collected, and then organs such as the heart, liver, spleen, lungs, kidneys, ovaries and uterus were washed, weighted and fixed in 4% paraformaldehyde.

### 2.3. H&E Staining

After mice were sacrificed, the heart, liver, spleen, lungs, kidneys, ovaries and uterus were fixed in 4% paraformaldehyde (Servicebio, Wuhan, China) for 24 h. Then, paraformaldehyde was removed before samples were dehydrated, cleaned, waxed and embedded in paraffin. Lastly, the specimens were sliced at a 5 µm thickness and stained by H&E staining.

### 2.4. Behavioral Tests

Here, we mainly tested the behaviors related to mouse emotion, including open field and elevated plus maze tests, considering the impact of the environment on the behavior of mice; all behavioral tests were performed from 9:00 a.m.–5:00 p.m.

#### 2.4.1. Open Field Test

In order to evaluate the locomotion and exploration abilities of mice treated by 150 mT static magnetic fields for 18 weeks, a nine-squared grid in the middle of the board was defined as the central area, and the others were defined as the peripheral area. We put the mice in the center area, and allowed them to freely explore for 6 min. The ANY-Maze video tracking system can automatically record and analyze distance, entries and the amount of time of mice spend in corresponding areas. To avoid contamination caused by previous mice, we cleaned the device with 75% ethanol after each test.

#### 2.4.2. Elevated Plus Maze Test

The elevated plus maze test is another device which can evaluate mice’s anxiety. Firstly, the device was wiped with 75% alcohol, and then mice were placed in the center of cross at the intersection of two horizontally closed arms and two vertically open arms. Mice were allowed to explore freely for 5 min, and entries and time in the open arms were recorded by the ANY-Maze Video Tracking System.

### 2.5. Enzyme-Linked Immunosorbent Assay (ELISA)

We took kits (Sinobest Biotechnology, Shanghai, China) from the 4 °C refrigerator to bring to room temperature 20 min in advance. We set blank, standard and sample wells, (blank wells were empty), added 50 μL of different concentrations of standard to each standard well, and then added 50 μL fresh serum to each sample well. A total of 100 μL of horseradish peroxidase (HRP)-labeled detection antibody was added to each well (except for blank wells), and the wells were then sealed with film and incubated at 37 °C. After 1 h, the liquid was discarded each well was filled with 350 μL washing solution, left to stand still for 1 min, and then the solution was shaken off, patted dry and repeated 5 times. We then added 50 μL of substrate A and 50 μL substrate B to each well, which were then incubated at 37 °C in the dark; after 15 min, we added 50 μL of stop solution to each well, and measured the OD value at a 450 nm wavelength within 15 min.

### 2.6. Total Antioxidant Capacity Assay (ABTS)

The assay for total oxidative capacity in serum was performed according to the manufacturer’s instruction (Beyotime Biotechnology, S0119, Shanghai, China). Briefly, 20 µL of peroxidase solution was added to each well of the 96-well plate, and 10 µL of PBS or serum was then added to the control or the tested wells and mixed. Secondly, we added 170 µL of ABTS solution to each well, which were then mixed gently, incubated for 6 min at room temperature and the OD value was measured at 414 nM.

### 2.7. Lipid Peroxidation MDA Assay

The assay for total oxidative capacity in serum was performed according to the manufacturer’s instruction (Beyotime Biotechnology, S0131S, Shanghai, China). Briefly, we added 100 µL of PBS or serum to the control or the tested wells, respectively. Then, we added 200 µL MDA solution, which was then mixed. The solution was boiled 15 min at 100 °C, cooled and centrifuged at 1000× *g* for 10 min. Subsequently, we added 200 μL of supernatant to a 96-well plate and measured the OD value at 535 nm.

### 2.8. Microbiota Analysis by 16S Sequencing

Total genome DNA from feces samples were extracted through OMEGA Soil DNA Kit (D5625-01) (Omega Bio-Tek, Norcross, GA, USA) according to the manufacturer’s protocols. The V3-V4 region of the prokaryotic 16S rDNA was amplified with forward primers containing the sequence 338F 5′-ACTCCTACGGGAGGCAGCA-3′ and reverse primers containing the sequence 5′-GGACTACNVGGGTWTCTAATCC-3′. Sample-specific 7-bp barcodes were incorporated into the primers for multiplex sequencing. PCR amplicons were purified with Vazyme VAHTSTM DNA Clean Beads (Vazyme, Nanjing, China) and quantified using the Quant-iT PicoGreen dsDNA Assay Kit (Invitrogen, Carlsbad, CA, USA). After the individual quantification step, amplicons were pooled in equal amounts, and pair-end 2 × 250 bp sequencing was performed using the Illlumina MiSeq platform with the MiSeq Reagent Kit v3 at Shanghai Personal Biotechnology Co., Ltd. (Shanghai, China). Microbiome bioinformatics were performed according to the official tutorials (https://docs.qiime2.org/2019.4/tutorials/ accessed on 10 July 2022). Alpha-diversity metrics and beta diversity metrics were estimated using the diversity plugin with samples, which were rarefied sequences per sample. Sequence data analyses were mainly performed using QIIME2 and R packages (v3.2.0). Microbial functions were predicted by PICRUSt2 upon MetaCyc (https://metacyc.org/ accessed on 5 August 2022) and KEGG (https://www.kegg.jp/ accessed on 5 August 2022) databases.

### 2.9. Statistical Analysis

All the data were expressed as mean ± SEM and analyzed using the Shapiro–Wilk test, and they all conformed to normal distribution. If the data was normal distribution, one-way ANOVA was used to evaluate the difference among groups by GraphPad Prism 9.4.1. Otherwise, the Mann–Whitney U test was used, and *p* < 0.05 was considered as statistically significant.

For the accuracy of the experimental results, we performed the analysis in a blind way, and the person who analyzed the data did not know the exposure conditions of the mice.

## 3. Results

### 3.1. Long-Term 150 mT SMFs Exposure Did Not Affect Quality of Life of Female Mice

**Figure 2:**
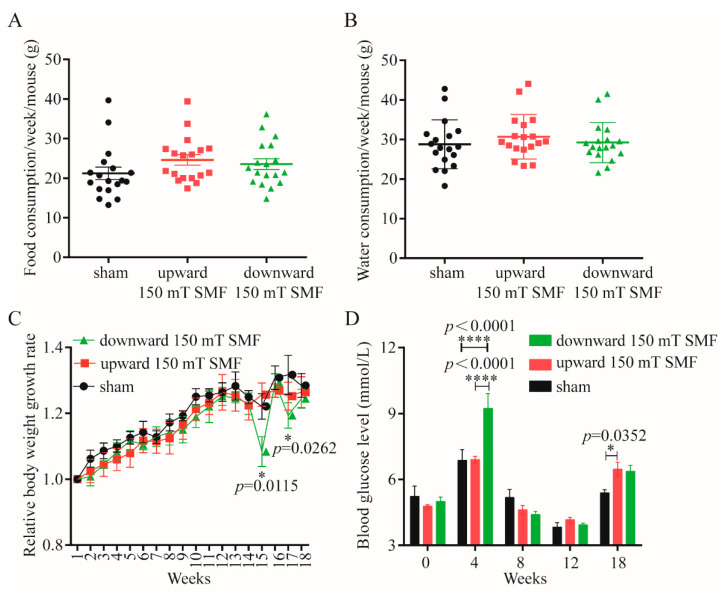
Effect of long-term 150 mT SMFs exposure on food and water intake, body weight and blood glucose in the female mice. Food (**A**) and water (**B**) consumed weekly by every mouse. (**C**) Relative body weight increased weekly. (**D**) Blood glucose of mice exposed to sham and 150 mT SMFs. At week 4, the bedding contained food residues, and fasting led to high blood glucose. We did statistical analysis for all data, and values show mean ± SEM, and “*” means *p* < 0.05, “****” means *p* < 0.0001. In consideration of the readability of the paper, no statistical significance (*p* > 0.05) was not labeled uniformly.

In order to explore the effect of long-term 150 mT SMF exposure on adult female mice, we measured the body weight, food and water consumptions on a weekly basis. Our data showed that there was no difference in food and water intake among upward and downward 150 mT SMFs compared with the sham group (Figure 2A,B); in fact, the food consumption upwards of 150 mT SMF (24.62 ± 5.57 g) was more than the sham group (21.21 ± 6.45 g). However, the weight of the upward 150 mT SMF group was lighter than the sham group (Figure 2C), which had no statistical significance. Except for these, we also detected blood glucose and found that blood glucose in upward (6.47 ± 0.71 mmol/L, *p* = 0.0352) and downward 150 mT SMFs (6.37 ± 0.60 mmol/L, *p* > 0.05) were both higher than the sham (5.38 ± 0.33 mmol/L).

**Figure 3:**
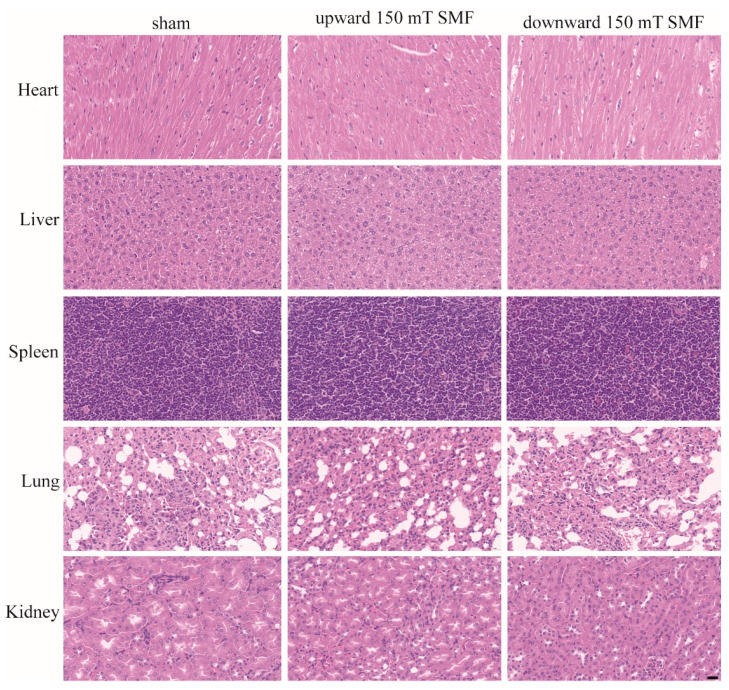
H&E staining showed that long-term 150 mT SMFs exposure did not cause significance damage to the major organs in female mice. Scale bar: 20 μm.

To further analyze long-term 150 mT SMFs exposure-induced physiological consequences in female mice, we performed a blood routine (Table 1) and biochemical analysis (Table 2). The results showed that long-term 150 mT SMFs exposure did not affect any physical parameters (Table 1) or organ functioning in female mice (Table 2), except for in the case of uric acid (UA) of the downward 150 mT SMF (99.95 ± 14.21 μmol/L, *p* = 0.0259) which was lower than the sham group (120.04 ± 8.02 μmol/L). The level of UA is related to metabolism and renal function, whereas the result of a complete blood count showed there was no significant difference between downward 150 mT SMF and the sham group (Table 1). Considering security, we weighed the main organs (heart, liver, spleen, lungs, kidneys, ovaries and uterus) of the mice and calculate the relative weight index after mice were sacrificed, and found there were no obvious differences between upward/downward 150 mT SMFs and the sham group (Table 3). Moreover, H&E staining of the organs (heart, liver, spleen, lungs and kidneys) also did not show obvious abnormalities for all mice (Figure 3).

### 3.2. Long-Term 150 mT SMFs Exposure Enhanced Locomotive and Exploratory Activity in Adult Female Mice

**Figure 4:**
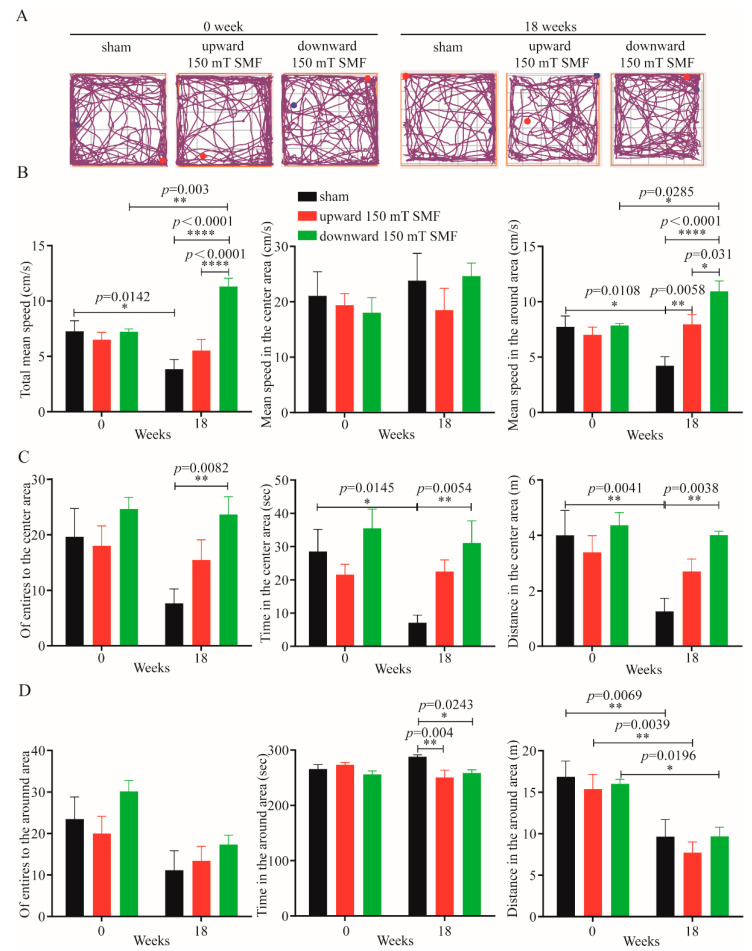
Open field tests showed that long-term 150 mT SMFs exposure could improve athletic ability and reduce anxiety-like behavior. (**A**) Movement trajectories of mice in open field experiments; the start and end points of the movement are indicated by the blue and red dots, respectively. (**B**) The mean movement speed of mice in sham and 150 mT SMF groups, including the mean speed in the central, peripheral area and the mean speed during the test. (**C**) Motion parameters of the mice in the central area, including the entries, time and distance. (**D**) Motion parameters of the mice in the around area, including the entries, time and distance. All the data were represented as the mean ± SEM. “*” means *p* < 0.05, “**” means *p* < 0.01, “****” means *p* < 0.0001.

To evaluate the effects of long-term 150 mT SMFs exposure on adult female mice, we performed open field and elevated plus maze tests. Firstly, we measured the locomotive and exploratory activities with an open field test (OFT) (Figure 4). The movement track was recorded for every mouse by one researcher (Figure 4A), and videos were analyzed in a blind way by another researcher. The speed and distance can reflect the locomotive activity (Figure 4B). These data showed that the older the mice, the lower the exercise capacity;, for example, the total average speed of the sham group ranged from 7.27 ± 2.13 cm/s, before being reduced to 3.85 ± 1.93 cm/s (reduced by 47.04%, *p* = 0.0142) after 18 weeks (Figure 4B). The distance in the center area moved and was reduced by 68.5% (*p* = 0.0041) (Figure 4C). It is interesting that long-term 150 mT SMFs exposure can attenuate the reduction and enhance locomotive and exploratory activity of the mice, especially downward 150 mT SMF. In fact, the movement of the downward speed of 150 mT SMF was increased by 65.99% (*p* < 0.0001) (Figure 4B) and distance in the center area was increased by 68.58% (*p* = 0.0038) compared with the sham group (Figure 4C). However, upward 150 mT SMF can also enhance the locomotive and exploratory activity of mice, but the effect was not as significant as the downward speed (Figure 4).

### 3.3. Long-Term 150 mT SMFs Exposure Reducse Anxiety- and Depression-like Behavior in Adult Female Mice

OFTs can not only reflect the locomotive and exploratory activity in mice, but also reflect anxiety and depression in mice. The number of entries and time spent in the center area reflected the anxiety of mice, as they naturally prefer to be stay within an area when they are depressed. The data showed that the older the mice, the shorter the time they spend in the center, and time spent in the center area from 28.55 ± 14.76 s was reduced to 7.1 ± 5.07 s (reduced by 75.13%, *p* = 0.0145) (Figure 4C). However, after downward long-term 150 mT SMF exposure, the number of entrances to the center area increased by 67.0% (*p* = 0.0082), and time in the center area was increased by 77.12% (*p* = 0.0054) compared to the sham group (Figure 4C).

To further determine the effects of long-term 150 mT SMFs exposure on the emotion of mice, we performed the elevated plus maze test, which can also reflect anxiety in mice. Mice preferred to stay in the two closed arms area and explore the open arms area occasionally in the elevated plus maze test (Figure S1A), so the time spent in the open arms and the times of entering the open arms are indicators of anti-anxiety levels. With older mice, the time spent in open arms was reduced from 76.62 ± 19.58 s to 40.03 ± 35.20 s (reduced by 47.76%, *p* > 0.05) in the sham group, whereas the time was increased from 85.85 ± 47.11 s to 92.73 ± 60.63 s (increased by 8.01%, *p* > 0.05) in the downward 150 mT SMF, although it had no statistical significance (Figure S1C). After 18 weeks, compared the downward 150 mT SMF with the sham group, not only did the number of mice entering the open arms increase by 13.85% (*p* > 0.05) (Figure S1B), but also the time spent in the open arms was increased by 131.65% (*p* > 0.05) (Figure S1C). Therefore, long-term 150 mT SMFs exposure had the potential to reduce anxiety- and depression-like behavior in adult female mice.

### 3.4. Long-Term 150 mT SMF Exposure Improved the Function of the Ovaries and Uterus in Adult Female Mice

**Figure 5:**
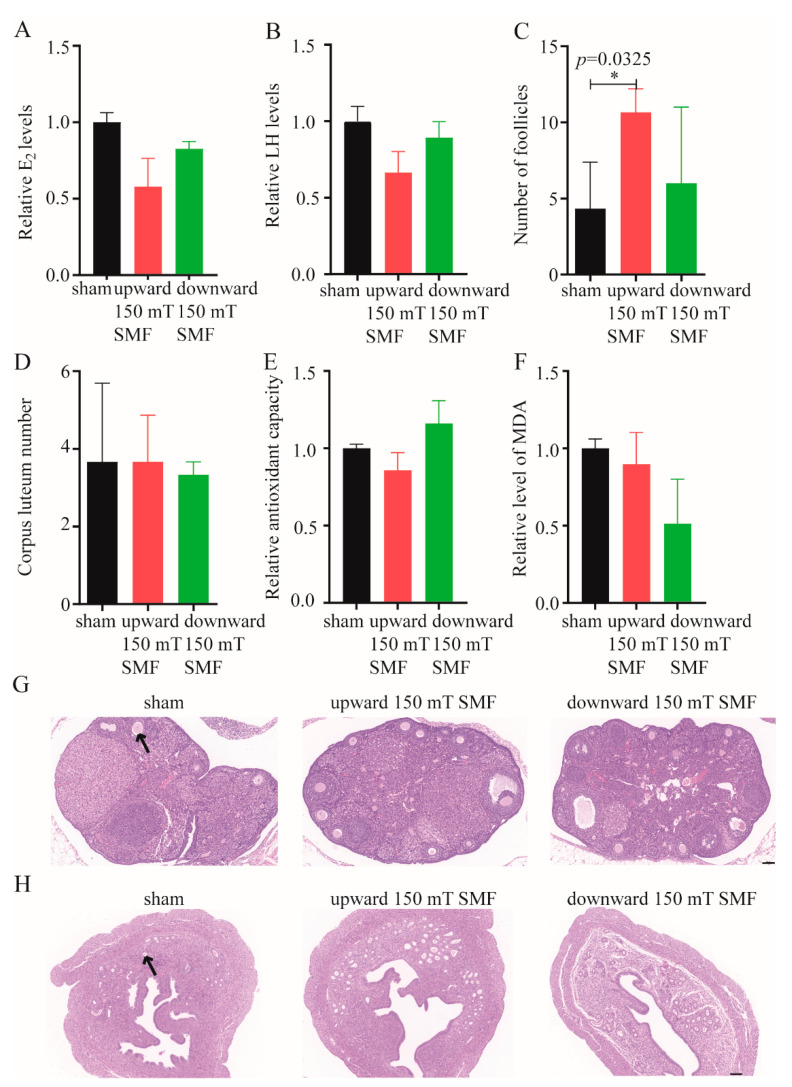
Effects of long-term 150 mT SMFs exposure improved the function of the ovaries and uterus in female mice. ELISA detected the E_2_ (**A**) and LH (**B**) levels of mice that were exposed to sham and 150 mT SMFs for 18 weeks. Statistics on the number of follicles (**C**) and corpus luteum (**D**) in the mice that were treated for 18 weeks. Relative total antioxidant capacity (**E**) and MDA level (**F**) of mice were detected after long-term exposure of 150 mT SMFs and sham. Representative H&E staining showed the effects of long-term 150 mT SMFs exposure on ovarian (**G**) and uterine (**H**) structures in female mice, the arrows point to the follicles (**G**) and uterine glands (**H**) respectively. Scale bar: 100 μm. We conducted statistical analysis for all data, and values show mean ± SEM, and “*” means *p* < 0.05. In consideration of the readability of the paper, no statistical significance (*p* > 0.05) was not labeled uniformly.

Women also have another very important function—reproduction, which is closely related to the hormone levels and ovarian and uterine function. It is well known that estrogen varies greatly in different menstrual cycles of females, and our results showed that the level of estradiol decreased by 43.8% (*p* = 0.0379) (Figure 5A), and the luteinizing hormone reduced by 35.2% (*p* = 0.0063) (Figure 5B) after upward long-term 150 mT SMF exposure. The ovaries are the main organ that secrete estrogen, and here, we found that long-term 150 mT SMF exposure increased the number of follicles and improved ovarian function, especially in the upward 150 mT SMF group (~2.5 times, *p* = 0.0325) (Figure 5C,G). Additionally, the results also showed that 150 mT SMFs increased the number of uterine glands and improved the function of the uterus (Figure 5D,H), which conceives the fetus and produces menstruation. For these reasons, we found that these changes were mainly attributed to long-term 150 mT exposure, and increased the total antioxidant capacity (Figure 5E) and decreased the level of lipid peroxidation (Figure 5F) in mice.

### 3.5. Long-Term 150 mT SMF Exposure Regulated Gut Microbiota and Metabolism in Adult Female Mice

**Figure 6:**
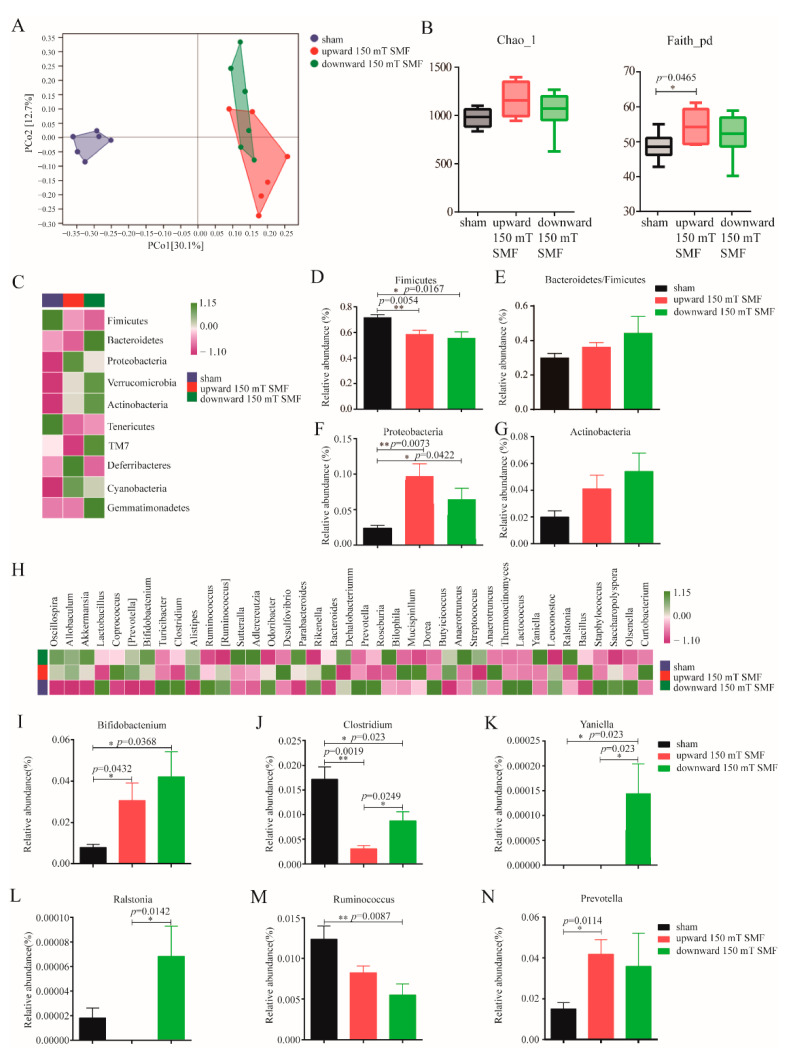
Long-term 150 mT SMF exposure regulated gut microbes’ population in adult female mice. (**A**) Principal coordinates analysis. (**B**) Species richness and diversity analyzed by Chao1 and Faith_pd in mice. (**C**) The taxonomic composition distribution at the phylum level in Top 10 (average value within the group). (**D**–**G**) The relative abundance of the ratio of *Proteobacteria*, *Actinobacteria*, *Verrucomicrobia* and *Bacteroidetes*/*Firmicutes* phyla. (**H**) The taxonomic composition distribution at the genus level in Top 40 (average value within the group). (**I**–**N**) The relative abundance of generea *Bifidobacterium*, *Clostridiaceae_Clostridium*, *Yaniella*, *Ralstonia* and *Ruminococcus*. “*” means *p* < 0.05 and “**” means *p* < 0.01.

By comparing the three groups, we found that both upward and downward 150 mT SMFs had significant beneficial effects on alleviating anxiety- and depression-like symptoms in adult female mice. To explore the unveiled mechanism, after 150 mT SMFs exposure, we collected and analyzed fecal samples that were directly related to exercise and emotions, and the degree of species accumulation and homogeneity analysis showed that this test was adequately sampled (Figure S2A,B). Interestingly, both upward and downward 150 mT SMFs exposure resulted in significant changes in overall gut microbiota structure, and increased species richness and diversity, as indicated by Chao1 and Faith_pd indices in adult female mice (Figure 6A,B). Compared with the sham group, 150 mT SMFs exposure increased the *Proteobacteria* (*p* < 0.05), *Actinobacteria*, *Verrucomicrobia* and *Bacteroidetes*/*Firmicutes* ratio, but reduced *Firmicutes* at the phylum level (*p* < 0.05) (Figure 6C–G and Figure S3). Moreover, at the genus level, *Bifidobacterium* abundance was significantly increased, and *Clostridiaceae_Clostridium* abundance was decreased in both upward and downward 150 mT SMFs (*p* < 0.05). The downward SMF also stimulated the growth of *Yaniella* (*p* < 0.05), *Ralstonia* (*p* < 0.05) and *Ruminococcus* (*p* < 0.01), but not the upward 150 mT SMF. In addition, the upward 150 mT SMF significantly increased *Prevotella* abundance (*p* < 0.05), but not the downward 150 mT SMF (Figure 6H–N and Figure S3).

**Figure 7:**
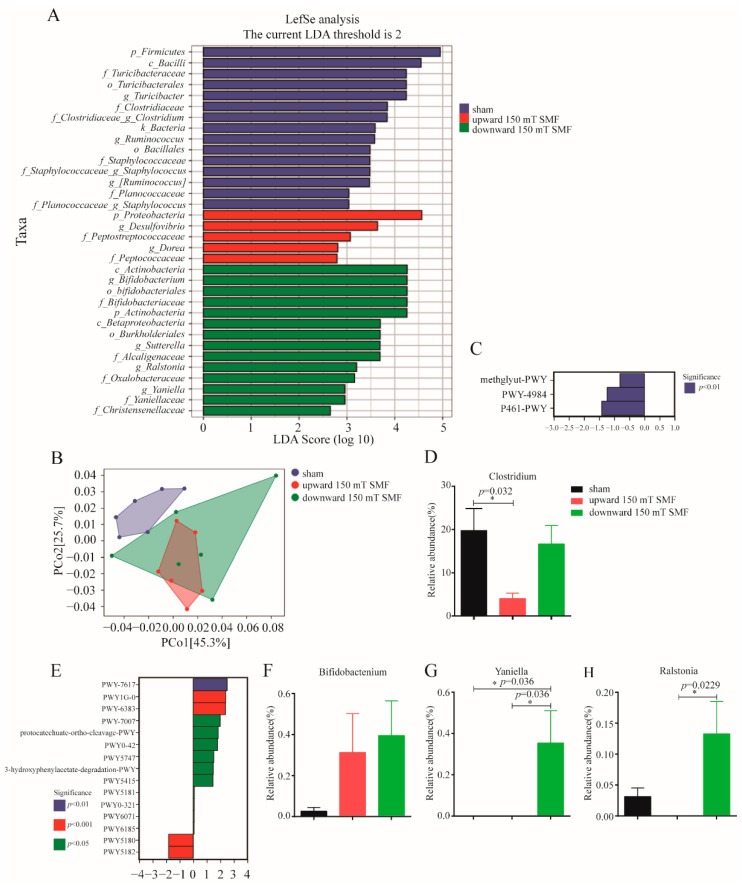
Long-term 150 mT SMFs exposure regulated metabolism function in adult female mice. (**A**) Histogram generated from LEfSe analysis exhibits differential microbes’ population in magnetic field treatment. Taxa enriched in microbiota from the Sham (blue), upward 150 mT SMF (red) or downward 150 mT SMF (green) groups were indicated with a positive LDA score, respectively (taxa with LDA score = 2 and significance of α < 0.05 determined by Wilcoxon signed- rank test). (**B**) PCoA analysis. (**C**) Metabolic pathway differences analysis between the upward 150 mT SMF and sham groups in adult female mice including methglyut-PWY, PWY-4984 and P461-PWY. (**D**) The relative abundance of genus *Clostridium* in PWY-4984. (**E**) Metabolic pathway differences analysis between the downward 150 mT SMF and sham groups in adult female mice. (**F**–**H**) The relative abundance of genus *g_Bifidobacterium* abundance *in* PWY1G-0, *g_Ralstonia* and *g_Yaniella* abundance in protocatechuate-ortho-cleavage-PWY and PWY-5180. “*” means *p* < 0.05.

To detect the specific bacteria that covaried with different SMF directions, linear discriminant analysis coupled with effect size (LEfSe) was subsequently employed. Obviously, *p_Proteobacteria*, *g_Desulfovibrio* and *g_Dorea* were associated with upward 150 mT SMF. Moreover, *p_ Actinobacteria*, *g_ Bifidobacterium*, *g_Sutterella*, *g_ Ralstonia* and *g_Yaniella* were associated with downward 150 mT SMF (Figure 7A and Figure S4). Interestingly, a PCoA analysis indicated that both upward and downward 150 mT SMFs resulted in significant changes in gut microbiota function, and the downward 150 mT SMF had a more extensive metabolic function than upward in adult female mice (Figure 7B). Obviously, compared with sham, the upward 150 mT SMF significantly reduced the metabolic functions of gut microbes, including methglyut-PWY, PWY-4984 and P461-PWY (*p* < 0.01) in adult female mice by metabolic pathway differences analysis. Moreover, analysis of metabolic pathway species composition revealed that the PWY-4984 metabolic pathway reduced the number of *Clostridium* (*p* < 0.05) and *Staphylococccus* (*p* < 0.01), indicating a reduction in anxiety symptoms in adult female mice (Figure 7C,D). In addition, downward 150 mT SMF significantly improved the metabolic function of gut microbes, including in PWY1G-0 (*p* < 0.001), PWY-6383 (*p* < 0.001), protocatechuate-ortho-cleavage-PWY and PWY-5415 (*p* < 0.05). In contrast, PWY-5180 and PWY-5182 (*p* < 0.001) decreased gut microbial metabolic function compared with the sham group, as analyzed by metabolic pathway species composition. Further analysis of metabolic pathway species composition revealed that increasing *g_Bifidobacterium*, *g_Ralstonia* and *g_Yaniella* abundance in downward 150 mT SMF exposure indicates that downward 150 mT SMF improved glucose metabolism and anxiety in adult female mice. (Figure 7E–H and Figure S5). Collectively, our data show that both upward and downward long-term 150 mT SMFs exposure can regulate microbiota redistribution in vivo.

## 4. Discussion

In this study, we found that long, 18-week exposure of 150 mT moderate intensity SMFs can enhance the locomotive activity and ameliorate bad emotions by regulating metabolism and gut microbiota in adult female mice.

Consistent with previous research, our results also showed that 150 mT moderate intensity SMFs have a similar effect to ultra-high or high SMF [10,13,16,19,22,23]. For example, Lv et al. found that time and distance in the center area, the number of entries to the center area and averaged velocity were reduced when compared immediately after 11.1–33.0 T 1 h exposure with sham in healthy male mice, whereas after a 2-week recovery, all these parameters changed from lower to higher [13]. Here, after a downward 150 mT SMF 18-week exposure, the time in the center area and number of entrances to the center area increased by 77.12% (*p* = 0.0054) and 67.0% (*p* = 0.0082), and the movement speed and distance in the center area of downward 150 mT SMF increased by 65.99% (*p* < 0.0001) and 68.58% (*p* = 0.0038) (Figure 4B) separately when compared with the sham group. Additionally, 150 mT SMF exposure had no negative effects unlike the 11.1–33.0 T 1 h exposure, reflecting the advantages of moderate intensity SMF, which apart from low cost, also has higher biosafety.

Finally, 150 mT long-term SMF exposure increased athletics in female mice; athletics requires energy, and glucose is the source of energy for mice. Previous research has shown that 10–128 mT SMF exposure increased the level of blood glucose [24,25,26,27,28,29]. Furthermore, our data has consistently shown the time-dependent effects of 150 mT SMF exposure on blood glucose, and raised blood glucose within normal range during the final weeks (Figure 2D). Athletics can not only alleviate depression, but can also regulate gut microbiota [10,30,31]. Clarkeet al. found that the gut microbiota of professional rugby players had greater alpha diversity and a higher relative abundance of 40 different bacterial taxa than lean sedentary controls [30]. Queipo-Ortuno et al. found a decrease in *Firmicutes* and increase in genus *Bifidobacterium* in a short voluntary wheel running exercise [32]. We also consistently found that long-term 150 mT SMF exposure decreased *Firmicutes* (reduced by 22.55%, *p* = 0.0167) and increased the genus *Bifidobacterium* (increased 5.33 times, *p* = 0.0368) (Figure 6D,I), especially in downward 150 mT SMF. *Firmicutes* is called the “fat bacteria”, because it absorbs excess calories from food and leads to obesity, and its reduction explained the lower body weight of mice in the 150 mT SMFs group compared with the sham group. *Bifidobacterium* is a probiotic and exercise can increase its abundance [32,33], and it was reported that *Bifidobacteria* improved athletic performance by improving anxiety and reducing stress in athletes [34]. Therefore, 150 mT SMFs ameliorate exercise capacity and gut microbiota, which in turn reduces the depression-like behavior in female mice.

Globally, females also play an important role—the breeder of life, a reality which is directly related to hormone levels and the ovaries. A previous study showed that the levels of gonadotropins (FSH and LH), progesterone, estrogen and ovarian weights were reduced after 6, 12 and 18 weeks of 50 Hz 25 mT magnetic field exposure, and these were harmful to mammalian fertility and reproduction [35]. Similarly, here, we found 150 mT SMFs, especially those upward in nature, significantly reduced the level of estradiol (42%, *p* > 0.05) and luteinizing hormone (34%, *p* > 0.0379), though there was no statistical significance (Figure 5A,B). In females, different estrogen levels at different menstrual cycles and estrogen level reduction is also a manifestation of premature ovarian failure. Bakacak et al. found that the number of ovarian follicles decreased significantly in rats exposed to a 900 MHz electromagnetic field for 15 days (15 min/d) (70.00 ± 19.03), compared with a sham group (150.25 ± 49.53) [36]. One year later, researchers found that prenatal, daily 1 h 900-MHz EMF exposure from day 13 to day 21 resulted in reduced ovarian follicle reservoirs in female pups at the onset of puberty [37]. Unlike with EMF, Jasmi et al. found that static magnetic field exposure resulted in greater resistance against injury of mice ovaries during the first step of the vitrification process [38]. Our data has consistently shown that long-term 150 mT SMFs exposure increased the total follicle number of female mice, especially in the upward 150 mT SMF group (~2.5 times compared with the sham group, *p* = 0.0325), but did not affect the number of corpora luteum (Figure 5C,D). Moreover, H&E staining also showed that long-term 150 mT SMFs exposure did not obviously damage the ovaries and uterus (Figure 5G,H). Further studies found that long-term 150 mT SMFs exposure increased total antioxidant capacity (Figure 5E), and this was consistent with previous reports. Sirmatel et al. found after 1.5 T SMF exposure, total antioxidant capacity (TAC) increased significantly in 33 male volunteers [39]. Along with the increase in total antioxidant capacity, the level of lipid peroxidation was reduced (Figure 5F) in mice, which was consistent with Öztürk’s study, which also found that MDA levels were reduced after 60 min, 100 mT SMF exposure in 24 non-cancerous adjacent gastric tissues [40].

## 5. Conclusions

Here, we report that long-term 150 mT SMF exposure not only enhanced the adult female mice’s locomotive activity, but also alleviated the mental state of adult female mice. We explored this mechanism and determined that long-term 150 mT SMF exposure can modulate glucose metabolism and gut microbiota and increase the level of beneficial flora and antioxidant capacity of the mice. This result suggests that moderate intensity long-term SMF exposure may be used a new way to ameliorate the locomotive and exploratory capacity and negative mood associated with female growth.

There is a great amount of data showing that moderate intensity SMFs have good safety and have many positive effects on human health. Therefore, we believe that in the future, moderate intensity SMFs can be used as a form of physical therapy for many chronic diseases such as insomnia, pain, mood disorders and female reproductive dysfunction. We also believe that the application of moderate intensity SMFs will be widely used in the near future, but a large number of clinical studies must be conducted before it can be widely used.

## Figures and Tables

**Table 1:** Complete blood count examination of long-term 150 mT SMFs and sham exposure. Comparisons were made between the sham and upward or downward 150 mT SMF groups Values show mean ± SEM, and statistical analysis was conducted for all data. In consideration of the readability of the paper, we did not label the data which had no statistical significance (*p* > 0.05).

Parameter	Sham (Mean ± SEM)	Upward 150 mT SMF (Mean ± SEM)	Downward 150 mT SMF (Mean ± SEM)
RBC (10^−12^/L)	9.67 ± 2.0	8.79 ± 1.49	8.93 ± 2.61
HCT (%)	47.20 ± 10.15	44.47 ± 7.41	44.40 ± 13.43
HGB (g/L)	139.83 ± 29.56	129.00 ± 23.06	132.17 ± 39.35
PLT (10^−9^/L)	780.5 ± 148.45	986.83 ± 233.42	904.33 ± 284.16
WBC (10^−9^/L)	5.60 ± 1.31	6.05 ± 2.12	4.17 ± 1.58
MCV (fL)	48.73 ± 0.75	50.68 ± 0.95	49.55 ± 0.91
Gran (%)	15.90 ± 1.65	14.32 ± 1.38	18.32 ± 2.75
MONO (%)	3.10 ± 0.42	2.70 ± 0.23	3.25 ± 0.79
Lymph (%)	81.00 ± 1.96	82.98 ± 1.49	78.43 ± 3.47

**Table 2:** Blood biochemical detection of mice exposed long-term to sham, upward and downward 150 mT SMFs. We conducted statistical analysis for all data; comparisons were made between each sham and 150 mT SMF group. Red marks illustrate that data was statistically more significant than sham. All groups contained six mice, and values show mean ± SEM, and “*” means *p* < 0.05. In consideration of the readability of the paper, no statistical significance (*p* > 0.05) was not labeled uniformly.

Blood Biochemical Analysis	Sham (Mean ± SEM)	Upward 150 mT SMF (Mean ± SEM)	Downward 150 mT SMF (Mean ± SEM)
ALT (IU/L)	39.52 ± 10.38	35.08 ± 5.32	31.74 ± 4.32
AST (IU/L)	192.43 ± 41.45	166.15 ± 55.76	147.60 ± 44.31
TBIL (μmol/L)	19.05 ± 4.47	17.11 ± 2.94	21.61 ± 5.17
UREA (mmol/L)	21.31 ± 4.29	25.48 ± 3.45	34.63 ± 14.45
CREA (mmol/L)	30.81 ± 3.81	29.61 ± 2.28	26.41 ± 2.63
UA (μmol/L)	120.04 ± 8.02	117.69 ± 10.79	99.95 ± 14.21 *
TG (mmol/L)	0.84 ± 0.26	1.13 ± 0.23	1.11 ± 0.54
TC (mmol/L)	2.92 ± 0.32	2.82 ± 0.24	2.93 ± 0.36
HDL-c (mmol/L)	1.36 ± 0.18	1.21 ± 0.08	1.20 ± 0.16
LDL-c (mmol/L)	0.42 ± 0.05	0.34 ± 0.08	0.37 ± 0.05

**Table 3:** Relative organ index of mice exposed long-term to sham, upward and downward 150 mT SMFs. We conducted statistical analysis for all data; comparisons were made between each sham and 150 mT SMF group. All groups contained six mice, and values show mean ± SEM. In consideration of the readability of the paper, no statistical significance (*p* > 0.05) was not labeled uniformly.

Relative Organ Index	Sham (Mean ± SEM)	Upward 150 mT SMF (Mean ± SEM)	Downward 150 mT SMF (Mean ± SEM)
Heart	0.0070 ± 0.0014	0.0071 ± 0.0009	0.0076 ± 0.0011
Liver	0.0444 ± 0.0050	0.0438 ± 0.0041	0.0450 ± 0.0051
Spleen	0.0046 ± 0.0006	0.0047 ± 0.0006	0.0049 ± 0.0006
Lung	0.0084 ± 0.0011	0.0082 ± 0.0005	0.0083 ± 0.0008
Kidney	0.0144 ± 0.0012	0.0137 ± 0.0006	0.0145 ± 0.0007
Ovarian + uterine	0.0110 ± 0.0026	0.0093 ± 0.0034	0.0092 ± 0.0024

## Data Availability

All data generated and analyzed during the current study are available from the corresponding authors on reasonable request.

## Supplementary Materials

The following supporting information can be downloaded at: https://www.mdpi.com/article/10.3390/biology11111585/s1, Figure S1: The elevated plus maze test showed that 4-month 150 mT exposure reduced the anxiety in female mice; Figure S2: Fecal samples were collected and analyzed after 150 mT SMFs long-term exposure; Figure S3: 150 mT SMFs long-term exposure regulated gut microbes’ population in adult female mice. Figure S4: 150 mT SMFs long-term exposure regulated metabolism function in adult female mice. Figure S5: Metabolic pathway differences analysis between the downward 150 mT SMF and sham in adult female mice.

## Abbreviations

SMF	Static magnetic field
SEM	standard error of mean
MRI	Magnetic resonance imaging
FDA	Food and drug administration
PBS	Phosphate-buffered saline
RBC	Red blood cell
HCT	Hematocrit
HGB	Hemoglobin
PLT	Platelet
WBC	White blood cell
MCV	Mean red blood cell volume
Gran	Granulocytes
MONO	Mononuclear cells
Lymph	Lymphocytes
ALT	Alanine transaminase
AST	Aspartate transaminase
UA	Uric acid
CREA	Creatinine
TBIL	Total bilirubin
TC	Total cholesterol
TG	Triglyceride
HDL-c	High-density lipoprotein cholesterol
LDL-c	Low-density lipoprotein cholesterol
OFT	Open field test
