# Supplementary figures and images for: The Effect of Long-Term Moderate Static Magnetic Field Exposure on Adult Female Mice
Source: Biology (Basel). 2022 Oct 28;11(11):1585. doi: 10.3390/biology11111585 (PMC9687991; doi:10.3390/biology11111585)

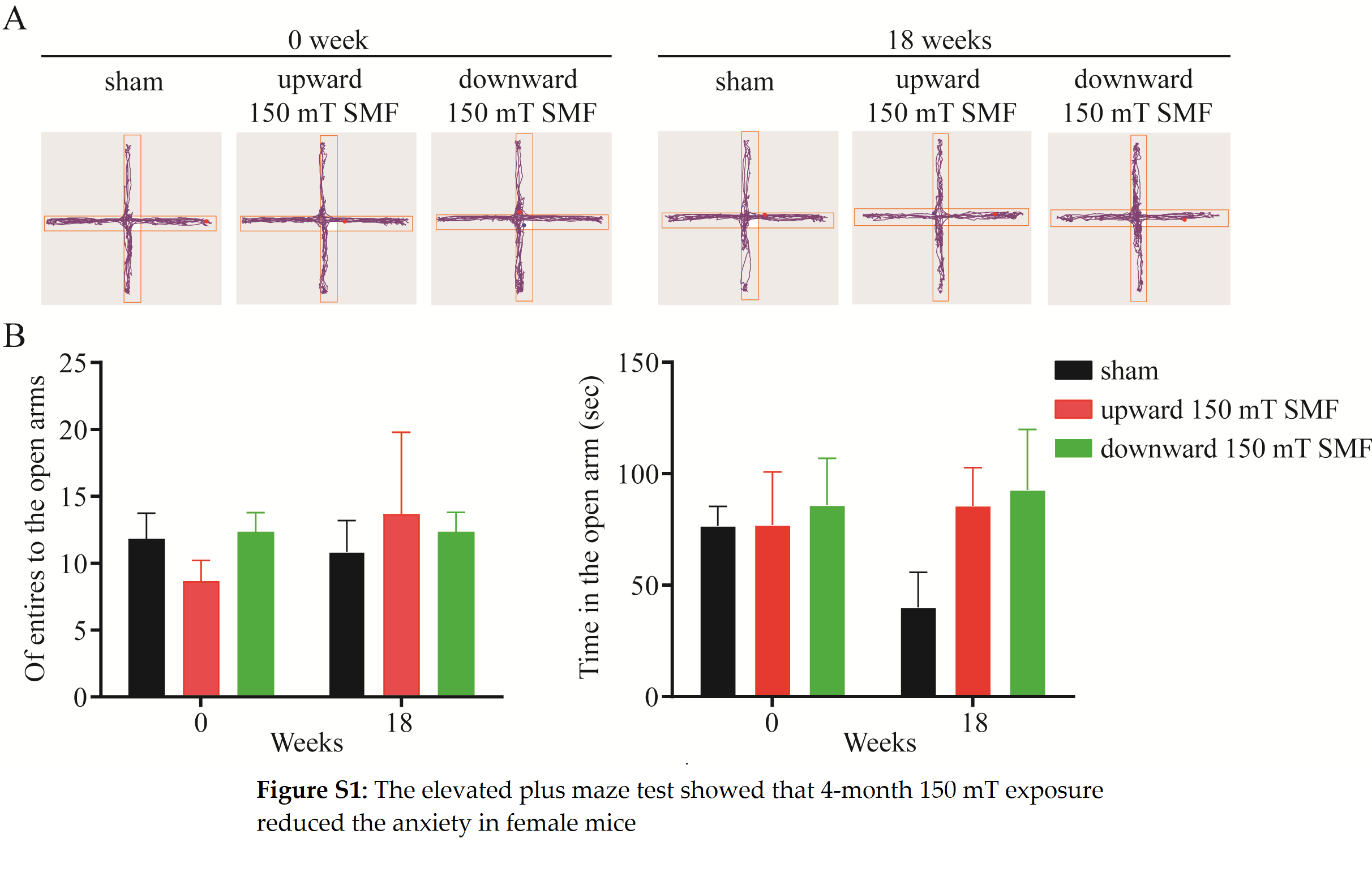

Supplement: Supplementary file 1 [file biology-11-01585-s001.zip › Figure S1.tif]

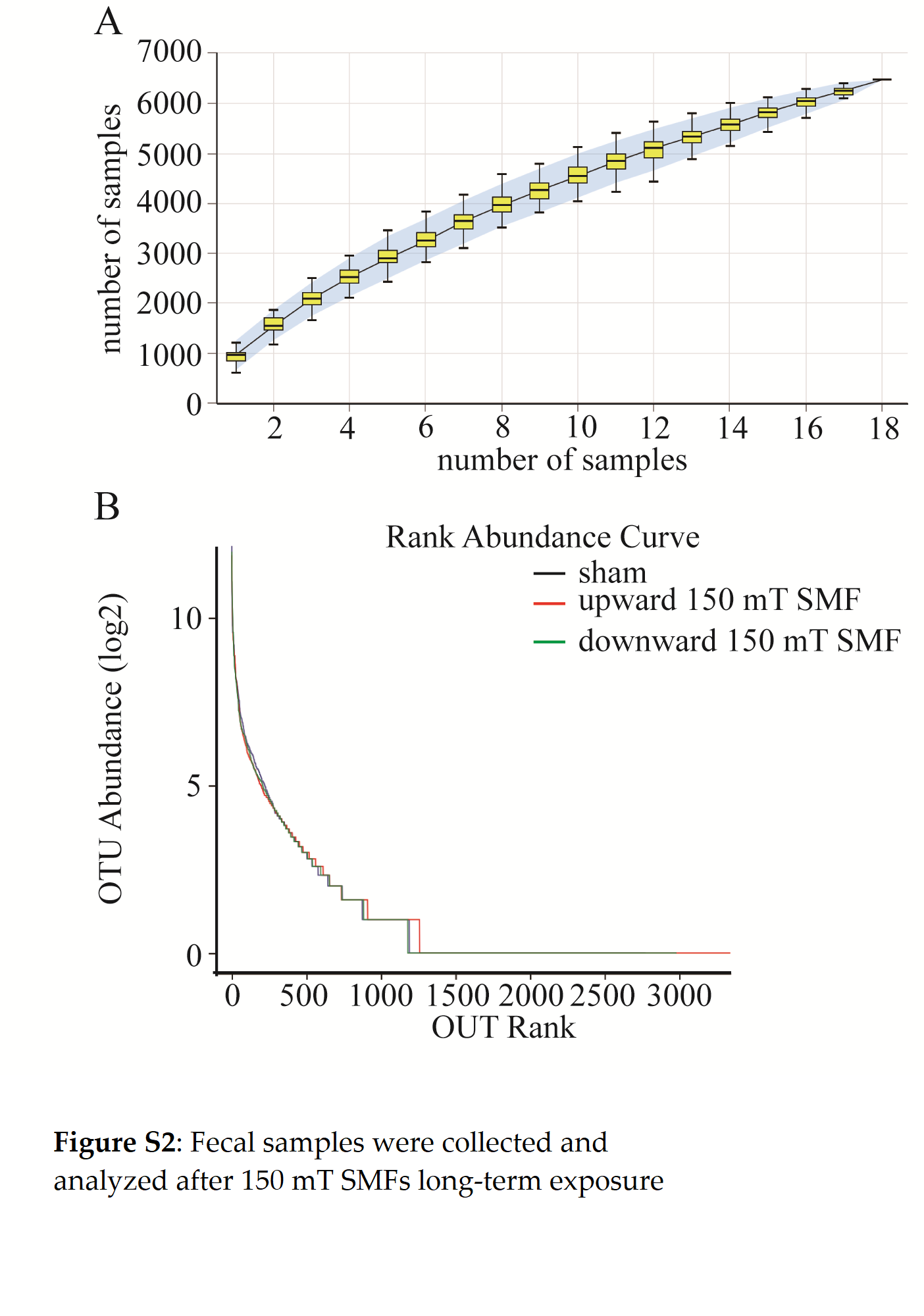

Supplement: Supplementary file 1 [file biology-11-01585-s001.zip › Figure S2.tif]

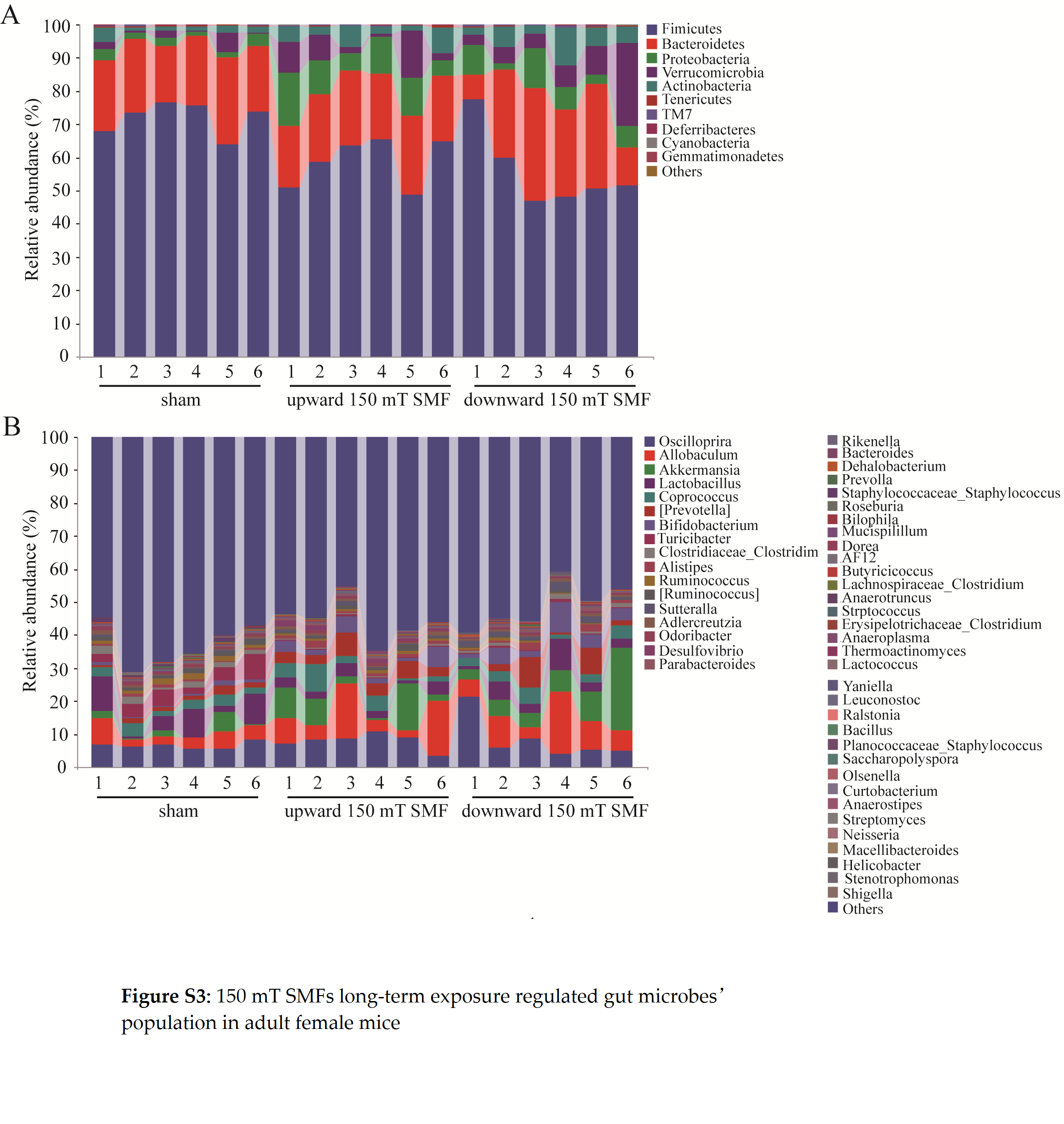

Supplement: Supplementary file 1 [file biology-11-01585-s001.zip › Figure S3.tif]

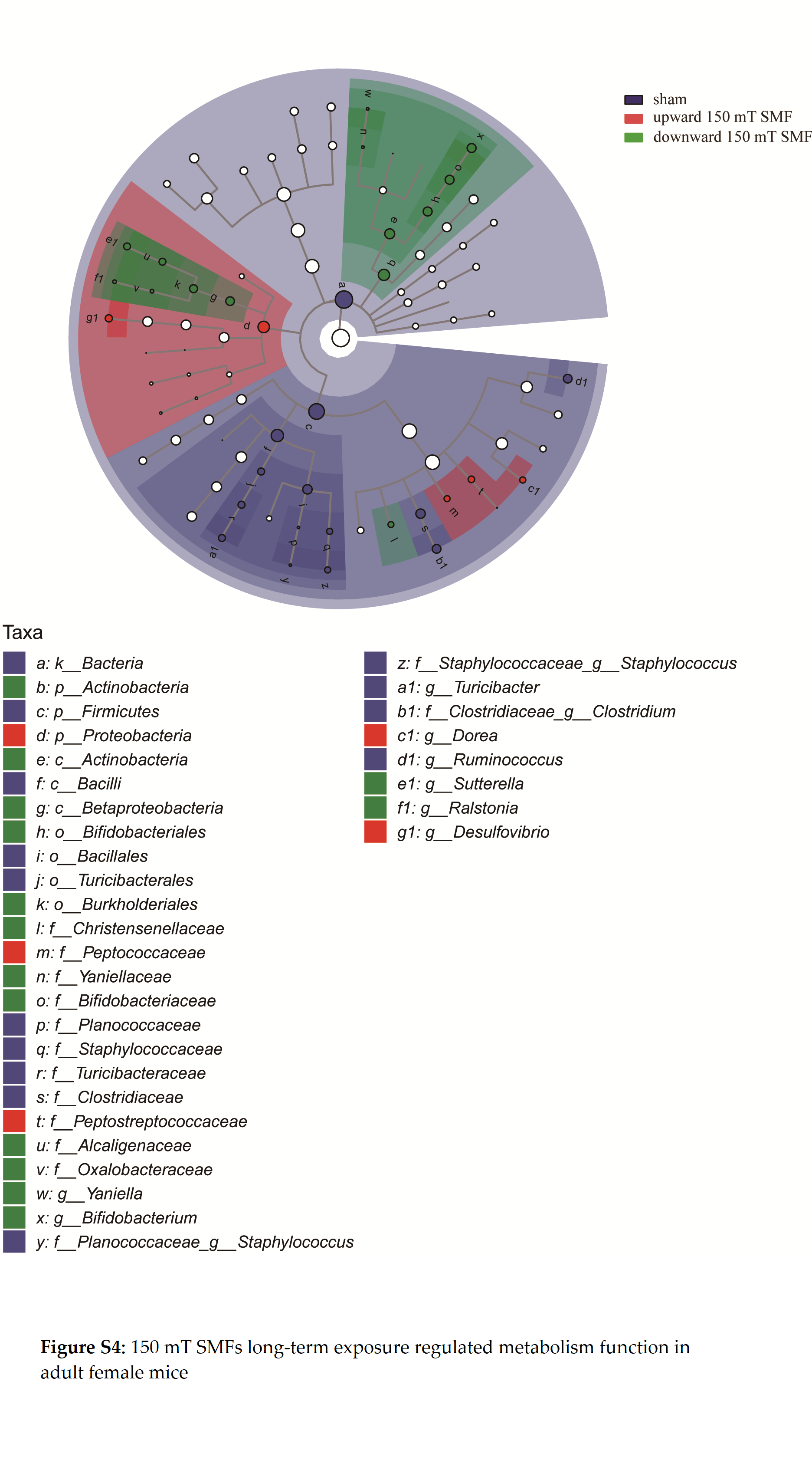

Supplement: Supplementary file 1 [file biology-11-01585-s001.zip › Figure S4.tif]

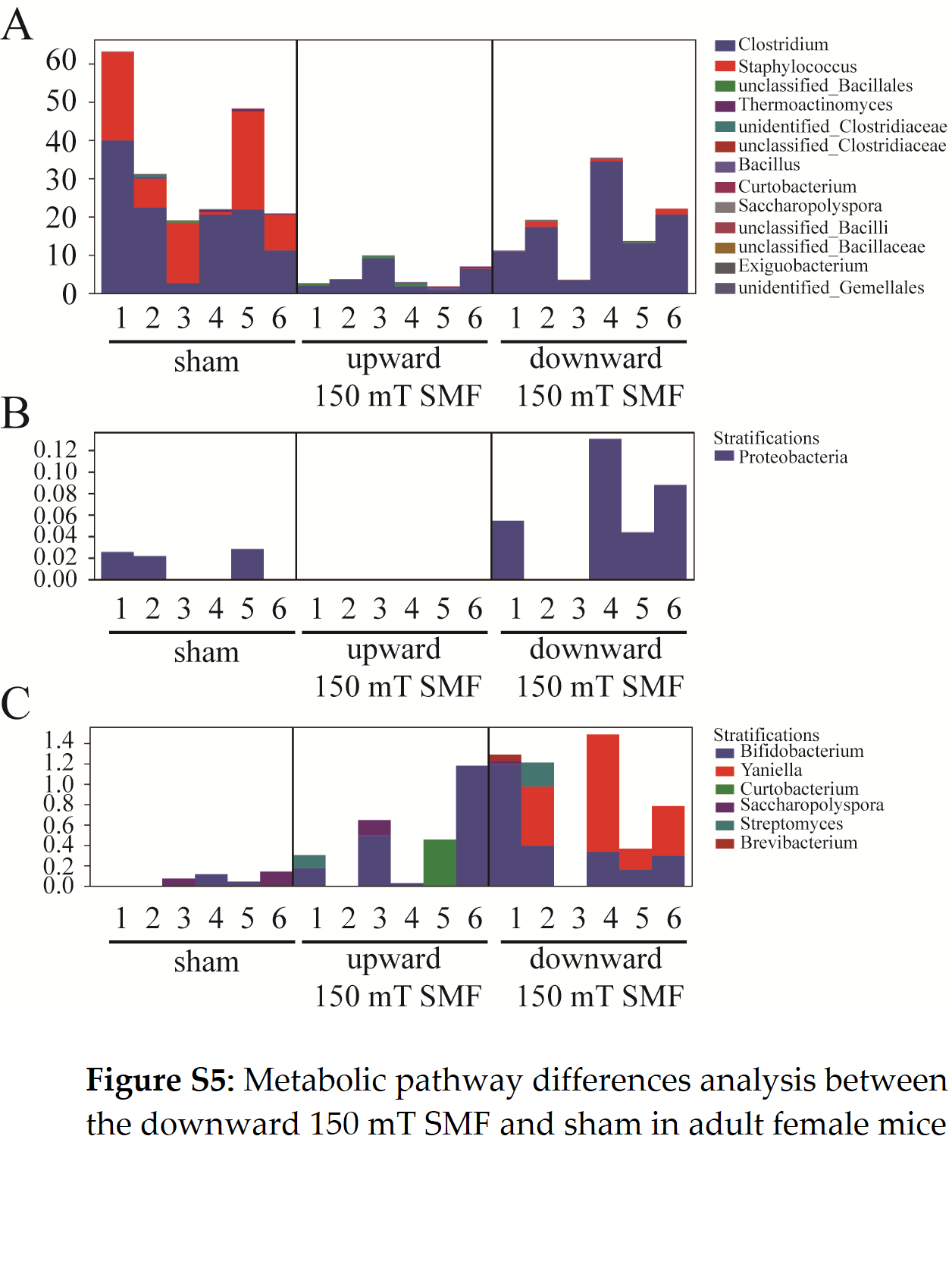

Supplement: Supplementary file 1 [file biology-11-01585-s001.zip › Figure S5.tif]
